# Collapse-to-emergency medical service cardiopulmonary resuscitation interval and outcomes of out-of-hospital cardiopulmonary arrest: a nationwide observational study

**DOI:** 10.1186/cc10219

**Published:** 2011-05-05

**Authors:** Soichi Koike, Toshio Ogawa, Senzan Tanabe, Shinya Matsumoto, Manabu Akahane, Hideo Yasunaga, Hiromasa Horiguchi, Tomoaki Imamura

**Affiliations:** 1 Department of Planning, Information and Management, The University of Tokyo Hospital, 7-3-1 Hongo, Bunkyo-ku, Tokyo 113-8655, Japan; 2 Department of Public Health, Health Management and Policy, Nara Medical University School of Medicine, 840 Shijocho, Kashihara, Nara 634-8521, Japan; 3 Foundation for Ambulance Service Development, Emergency Life-Saving Technique Academy of Tokyo, 4-5 Minami-osawa, Hachioji, Tokyo 192-0364, Japan; 4 Department of Health Management and Policy, Graduate School of Medicine, The University of Tokyo, 7-3-1 Hongo, Bunkyo-ku, Tokyo 113-8655, Japan

## Abstract

**Introduction:**

The relationship between collapse to emergency medical service (EMS) cardiopulmonary resuscitation (CPR) interval and outcome has been well documented. However, most studies have only analyzed cases of cardiac origin and Vf (ventricular fibrillation)/pulseless VT (ventricular tachycardia). We sought to examine all causes of cardiac arrest and analyze the relationship between collapse-to-EMS CPR interval and outcome in a nationwide sample using an out-of-hospital cardiac arrest (OHCA) registry.

**Methods:**

This was a retrospective observational study based on a nationwide OHCA patient registry in Japan between 2005 and 2008 (n = 431,968). We included cases where collapse was witnessed by a bystander and where collapse and intervention time were recorded (n = 109,350). Data were collected based on the Utstein template. One-month survival and neurologically favorable one-month survival were used as outcome measures. Logarithmic regression and logistic regression were used to examine the relation between outcomes and collapse-to-EMS CPR interval.

**Results:**

Among collapse-to-EMS CPR intervals between 3 and 30 minutes, the logarithmic regression equation for the relationship with one-month survival was y = -0.059 ln(x) + 0.21, while that for the relationship with neurologically favorable one-month survival was y = -0.041 ln(x) + 0.13. After adjusting for potential confounders in the logistic regression analysis for all intervals, longer collapse-to-EMS CPR intervals were associated with lower rates of one-month survival (odds ratio (OR) 0.93, 95% confidence interval (CI): 0.93 to 0.93) and neurologically favorable one-month survival (OR 0.89, 95% CI 0.89 to 0.90).

**Conclusions:**

Improving the emergency medical system and CPR in cases of OHCA is important for improving the outcomes of OHCA.

## Introduction

The recovery rate in patients suffering cardiopulmonary arrest is generally very low for out-of-hospital cases [1]. In spite of a substantial effort, studies have found that the overall survival in out of hospital cardiac arrest (OHCA) has been stable for almost 30 years [2], or has shown little improvement [3]. As such, establishing an effective emergency medical system (EMS) as well as improving the quality of basic life support (BLS) and advanced cardiac life support (ACLS) are important health policy issues. A number of previous studies have reported that starting cardiopulmonary resuscitation (CPR) earlier results in better outcomes, applying regression models [4], logistic regression models [5,6], and reciprocal models [7] to describe the relationship between collapse-to-EMS CPR interval and outcome.

This study examined the relationship between collapse-to-EMS CPR interval and outcomes based on a nationwide OHCA registry. As such, this study is one of the largest studies conducted, in terms of its study population and coverage. There is currently limited documentation on the effects of collapse-to-CPR interval on this scale. Most previous studies have analyzed cardiac origin only, especially initial rhythms of ventricular fibrillation (Vf) or pulseless ventricular tachycardia (VT), A nationwide analysis of all causes of OHCA could provide useful information for establishing more effective EMS systems and the most appropriate allocation of resources.

The aim of this study was to analyze the relationship between the collapse-to-EMS CPR interval, one-month survival, and neurologically favorable outcome using a nationwide OHCA registry between 1 January 2005 and 31 December 2008. This study sought used curve-fitting analysis and potential confounder adjusted odds ratios of the collapse-to-EMS CPR interval. In addition, we sought to discuss the implications of our results for improving EMS systems and the survival of OHCA patients.

## Materials and methods

### Study design

This study was an observational, retrospective study based on an analysis of a nationwide OHCA registry in Japan from January 2005 to December 2008.

### Setting

Japan is a country with a population of 126 million and universal health insurance coverage. The universal emergency access number enables direct connection to a dispatch center located in the regional fire defense headquarters. Upon receiving a call, the nearest available ambulance is sent to the incident. All expenses for transport are covered by the local government and there is no charge to the patient [7]. The emergency network covers the whole country and almost all OHCA patients undergo emergency transfer to a hospital. Treatment fees for medical services at a hospital are also covered by health insurance. The data used in this study were recorded based on the Utstein template [8]. Items included in the database were the patient's name, sex, age, time of collapse (the time at which sudden falling into unconsciousness was either seen or heard by a witness), the first documented cardiac rhythm, etiology, the CPR or first defibrillation time, the time to return of spontaneous circulation (ROSC), the one-month survival rate, and the one-month CPC (cerebral performance category; as a measure of neurologically favorable survival) [9,10]. Location of arrest, survival at discharge, neurological outcome at discharge were not stored in the database. Cardiac etiology was composed of confirmed and presumed cardiac etiology. Although we could not confirm that all times in the database were recorded with standardized timing methods, the proportion of EMS teams practicing daily clock synchronization increased from 39% in December 2005 to 43% in July 2007 [11]. These data were transferred from regional fire defense headquarters to the Fire and Disaster Management Agency. Time data were recorded in the system in the unit of minutes.

### Selection of participants

**Figure 1:**
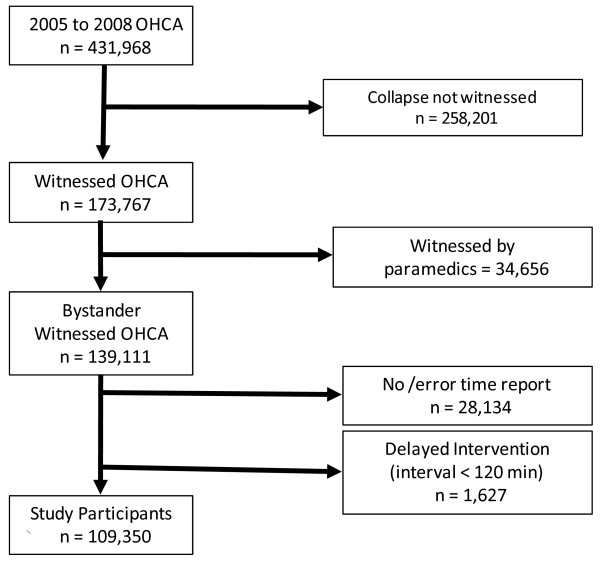
**Selection of study participants**.

Among the 431,968 OHCA emergency-transferred patients between January 2005 and December 2008, our analysis included cases where collapse was witnessed (that is, collapse was heard or seen by a bystander) but not witnessed by paramedics, the onset time was recorded, and intervention time was less than 120 minutes. A total of 109,350 cases were included in the analysis (Figure 1).

One-month survival was not recorded in 2,131 patients (1.9%) and neurologically favorable survival of 2,356 patients (2.2%) was not recorded in the data registry. These cases were excluded from the logistic regression analysis for outcome.

We obtained permission to analyze the data from the Fire and Disaster Management Agency of Japan, and the Agency provided an anonymized dataset. This study was approved by the Institutional Review Board of the Nara Medical University.

### Methods of measurement

Our primary outcome measurement was one-month survival. Neurologically favorable (CPC 1 (Good Cerebral Performance) or 2 (Moderate Cerebral Disability) was used as secondary outcome measurement. Etiology, one-month survival, and neurologically favorable one-month survival were recorded by EMS personnel in cooperation with attending physicians at medical institutions [12].

### Primary data analysis

After obtaining the patient characteristics and stratified outcome data, the relationship between collapse to EMS CPR interval and outcomes, logarithmic regression analyses were conducted for cases where collapse-to-EMS CPR time was between 3 and 30 minutes.

Logistic regression analyses where the dependent variable was one-month survival or neurologically favorable one-month survival and the independent variables were potential confounders including study year (2005 to 2006/2007 to 2008), sex (male/female), age (seven categories), etiology (cardiac origin/non-cardiac origin), bystander CPR (0/1), public Automated External Defibrillator (AED) (0/1) and collapse-to-EMS CPR interval (minutes) were then performed. In these logistic regression models, collapse-to-EMS CPR interval was treated as a continuous variable and included in the model as an independent variable. SPSS 16.0J (SPSS Japan Inc, Tokyo, Japan) was used for statistical analysis.

## Results

### Characteristics of study subjects

**Figure 2:**
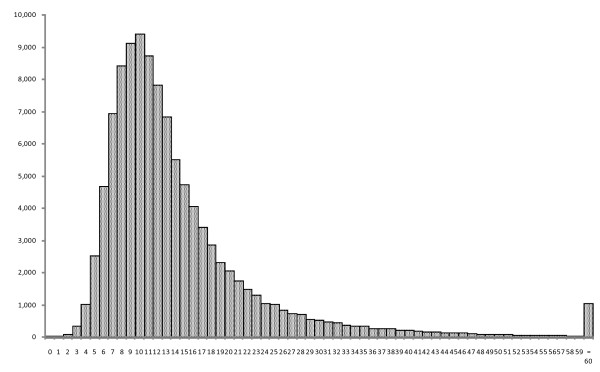
**Distribution of collapse-to-EMS CPR intervals (minutes)**. The distribution of patients by collapse-to-EMS CPR interval (minutes) was presented for 109,350 cases. Cases where the interval was equal or longer than 60 minutes were categorized into one group.

The characteristics of study participants are presented in Table 1. Among 109,350 study participants, 67,583 (61.8%) were male with mean age ± standard deviation (SD) of 72.9 ± 18.2 years old. The presumed etiology in 59,693 (54.6%) cases was cardiac origin, and non-cardiac origin in 49,657 (45.4%) cases. Bystander CPR was given in 49,122 (44.9%) cases, and 914 (0.8%) were treated by public AED. The mean collapse-to-EMS CPR interval (± SD) was 14.5 (± 9.3) minutes. The mean collapse-to-EMS CPR interval exhibited a positively skewed distribution (Figure 2). The other outcomes stratified by intervention or participant characteristics are presented in Table 2.

**Table 1:** Characteristics of study participants

Variable	No.(%) of patients	
Survey year		
2005	24,955	(22.8)
2006	26,861	(24.6)
2007	28,126	(25.7)
2008	29,408	(26.9)
	
Male sex	67,583	(61.8)
		
Age, mean (SD), year	72.9	(18.2)
		
Etiology		
Presumed cardiac	59,693	(54.6)
Non-cardiac	49,657	(45.4)
*cerebrovascular disease*	*5,331*	*(10.7)*
*respiratory diseases*	*7,041*	*(14.2)*
*cancer*	*3,982*	*(8.0)*
*exogenous causes*	*20,320*	*(40.9)*
*other non-cardiac origin*	*12,983*	*(26.1)*
*non-cardiac origin, subtotal*	*49,657*	*(100.0)*
		
Bystander CPR	49,122	(44.9)
*family*	*27,997*	*(57.0)*
*friend*	*2,202*	*(4.5)*
*colleague*	*1,610*	*(3.3)*
*passerby*	*1,767*	*(3.6)*
*others*	*15,546*	*(31.6)*
*type of bystander subtotal*	*49,122*	*(100.0)*
		
Public AED	914	(0.8)
Intubation	52,123	(47.7)
Drug	6,410	(5.9)
		
Interval, mean (SD), minutes		
collapse-to-call interval	5.4	(8.1)
collapse-to-arrival	12.8	(9.0)
collapse-to-EMS contact	14.0	(9.2)
collapse-to-EMS CPR	14.5	(9.3)
collapse-to-EMS defibrillation	16.7	(10.1)
collapse-to-hospital transfer	36.7	(14.5)
		

**Table 2:** One-month survival and neurologically favorable one-month survival

	One-month survival		Neurologically favorable one-month survival	
	No. (%) of patients		No. (%) of patients	
				
Survey Year				
2005 to 2006	3,758	(7.3)	1,545	(3.0)
2007 to 2008	5,269	(9.2)	2,803	(4.9)
				
Sex				
Male	6,087	(9.0)	3,134	(4.6)
Female	2,940	(7.0)	1,214	(2.9)
				
Age (year)				
<40	940	(13.3)	593	(8.4)
40 to 49	569	(12.1)	388	(8.3)
50 to 59	1,304	(12.7)	779	(7.6)
60 to 69	1,846	(11.1)	966	(5.8)
70 to 79	2,116	(7.6)	866	(3.1)
80 to 89	1,760	(5.9)	606	(2.0)
≥90	492	(3.8)	150	(1.2)
				
Etiology				
Non-cardiac	3,557	(7.2)	1,212	(2.4)
Presumed cardiac	5,470	(9.2)	3,136	(5.3)
				
Bystander CPR				
no bystander CPR	3,974	(6.6)	1,496	(2.5)
bystander CPR	5,053	(10.3)	2,852	(5.8)
				
Public defibrillation				
no public AED	8,414	(8.0)	3,927	(3.7)
public AED	343	(37.5)	296	(32.4)
				
Total	9,027	(8.3)	4,348	(4.0)

### Main results

**Figure 3:**
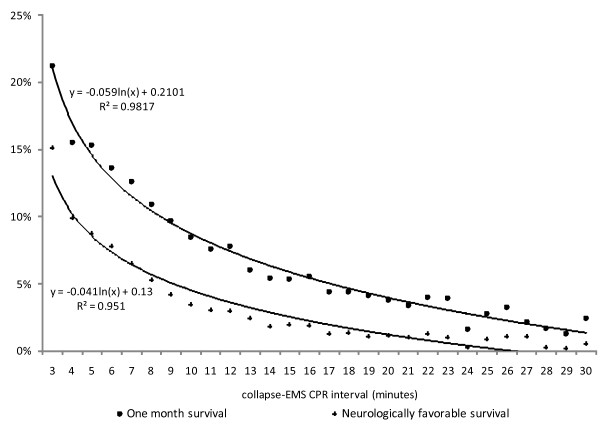
**Collapse-EMS CPR interval and outcomes**. The relationship between collapse-to-EMS CPR interval and one-month survival (dots) and neurologically favorable one-month survival (crosses) are presented for all cases where collapse-to-EMS CPR interval was between 3 to 30 minutes. Logarithmic regression equations for outcome (y) by collapse-to-EMS CPR interval (x) with R^2 ^were calculated and plotted in the graph.

Among cases where collapse-to-EMS CPR intervals (x) were between 3 and 30 minutes, the logarithmic regression equation for the relationship to one-month survival (y) was y = -0.059 ln(x) + 0.21 (R^2 ^= 0.98), and that with neurologically favorable one-month survival (y) was y = -0.041 ln(x) + 0.13 (R^2 ^= 0.95; Figure 3).

The results of the logistic regression analyses for one-month survival and neurologically favorable one-month survival revealed that the 2007 to 2008 period, male, cardiac origin, younger age, bystander CPR, public AED usage were all associated with higher rates of one-month survival and neurologically favorable one-month survival. After adjusting for the potential confounders presented above, the collapse-to-EMS CPR interval (minutes) was associated with lower survival (odds ratio (OR); 0.93, 95% CI (confidence interval); 0.93 to 0.93 (0.925 to 0.933)) and neurologically favorable one-month survival (OR; 0.89, 95% CI; 0.89 to 0.90; Table 3).

**Table 3:** Results of regression analysis

	One-month survival OR (95%)	Neurologically favorable one-month survival OR (95%)
***Survey year***		
2005 to 2006	Reference	Reference
2007 to 2008	1.16 (1.11 to 1.22)	1.41 (1.31 to 1.51)
***Sex***		
Male	Reference	Reference
Female	0.91 (0.87 to 0.96)	0.83 (0.77 to 0.90)
***Age (year)***		
<40	Reference	Reference
40 to 49	0.89 (0.79 to 1.01)	0.91 (0.78 to 1.07)
50 to 59	0.95 (0.86 to 1.05)	0.82 (0.72 to 0.94)
60 to 69	0.83 (0.75 to 0.92)	0.63 (0.56 to 0.72)
70 to 79	0.56 (0.52 to 0.62)	0.34 (0.30 to 0.39)
80 to 89	0.41 (0.37 to 0.45)	0.18 (0.15 to 0.20)
≥90	0.24 (0.21 to 0.27)	0.09 (0.07 to 0.11)
		
***Etiology***		
Non-cardiac origin	Reference	Reference
Cardiac origin	1.29 (1.23 to 1.35)	2.61 (2.41 to 2.84)
		
***Bystander CPR***		
No bystander CPR	Reference	Reference
Bystander CPR	1.49 (1.40 to 1.54)	1.95 (1.81 to 2.09)
		
***Public defibrillation***		
No public AED	Reference	Reference
Public AED	2.91 (2.44 to 3.47)	3.52 (2.88 to 4.31)
		
***Collapse-EMS CPR interval (minutes)***	0.93 (0.93 to 0.93)	0.89 (0.89 to 0.90)

CI, confidence interval; OR, odds ratio.

## Discussion

The present study was an analysis of data from 109,350 patients whose cardiac arrest onset was witnessed. Among cases where the collapse-to-EMS CPR interval was between 3 and 30 minutes, the duration of the collapse-to-EMS CPR interval was fitted to a logarithmic regression equation to examine its relationship with one-month survival and neurologically favorable one-month survival. After adjusting for potential confounders in a logistic regression analysis, we found that longer collapse-to-EMS CPR intervals were associated with lower one-month survival and neurologically favorable one-month survival.

Consistent with previous studies, the rate of one-month survival decreased sharply and gradually leveled off with increasing collapse-to-EMS CPR intervals. The nature of the relationship was the same after adjusting potential confounders including survey year, sex, age, etiology, bystander CPR and public AED. However, in previous studies, 20% to 34.1% [13-15] of cases were of non-cardiac origin, whereas the proportion of non-cardiac origin cases in the present study was 45.4%. This difference in etiological proportion should be considered when interpreting the results. The rate of survival following out-of-hospital cardiac arrest of non-cardiac origin has been previously reported to be lower than the survival rate in cases of cardiac arrest of cardiac origin [16]. Most previous studies limited the sample to cardiac origin only, De Mario *et al*. [17] analyzed all cardiac cases of arrest meeting the Utstein Criteria (9,273 patients) between 1991 and 1997, and confirmed that survival exhibited an exponential relationship with time. As our study has a much larger sample, our results provide additional evidence confirming the shape of the survival curve.

The shape of this survival curve suggests two ways to improve the survival of OHCA patients; shortening the collapse-to-CPR interval, or, alternatively, shifting the curve upward by improving the quality of resuscitation attempt.

To quicken response times, potential bystanders could be better educated to activate EMS as soon as possible. In addition, the ambulance system response could be streamlined, strengthening the "chain of survival" [18] concept and reinforcing the importance of an appropriate sequence of pre-hospital care. In Japan, the Fire and Disaster Management Agency reported that the mean response time (call-to-arrival interval) was 7.0 minutes in 2007, increasing from 6.1 minutes in 1997 [19]. In the same period, the number of traffic accidents and accompanying emergency transfers decreased. However, there has been a steady increase in the number of requests for ambulance services. The number of ambulance requests in Japan reached almost 5.3 million per year (almost a 50% increase in 10 years), but not all calls were genuine emergency cases. It was found that 51.7% of cases eventually did not require hospitalization. For fully utilizing limited resources in the most appropriate manner, the public should be better educated to call ambulance service only in case of an emergency. In addition, assessment and triage systems should be established at emergency control centers. These changes should be accompanied by improved transportation systems, including methods for determining the hospital to which the transfer should be made as rapidly as possible.

Starting CPR as early as possible would shift the survival curve left. In addition, the survival curve could be shifted upward by improving the quality of resuscitation attempts. High-quality CPR is a cornerstone of a system of care that can optimize outcomes [20]. It has been found that improved CPR quality administered by bystanders [21] and ACLS [22] are correlated with survival rates [23]. Various educational courses including mass CPR training and targeted CPR training for family members of patients suffering from cardiovascular diseases are currently available in Japan. Since 1995, new driver's license applicants have been required to take three hours of basic life support (BLS) training at driving schools [24], an attempt to expand BLS knowledge to the general public. Since 2003, Emergency Medical Technicians, (the highest level of ambulance personnel), have been authorized to use AED without online medical control. In the same year, orotracheal intubation was included as a sanctioned method of clearing airways by Emergency Life-Saving Technicians (ELSTs) with 262 hours of additional national standard training. Adrenaline administration by ELSTs with 220 hours of training became legal in 2006 [25]. These combined efforts to improve all four chains of survival could shift the survival curve upward, substantially improving the rate of survival in cases of OHCA.

Several limitations of this study should be considered. First, the time of collapse was based on interviews with laypersons. The witnesses might have been unable to accurately report the time of collapse. Unless there is an exceptional situation (for example, an OHCA event that is videotaped in a casino [26]), obtaining accurate collapse time is problematic, especially based on interviews with laypeople in emergency situations. Isaacs and colleagues [27] reported that layperson estimation of the time and actual measured intervals in cardiac arrest situations were not strongly correlated. As such, the quality of the time interval data represents a serious limitation of the current study. However, this limitation was minimized in the current analysis by excluding values that appeared to be due to error. In addition, the duration of the collapse-to-EMS CPR interval exhibited a positively skewed distribution, suggesting that the remaining potential errors in a set of 109,350 cases did not substantially affect the overall conclusions of this study.

A second limitation is that our data were obtained in Japan only. As such, the emergency system and demography might affect the results as unpredicted confounding factors. In our study, more than half of the study participants were 70 years old or older. It is known that the survival rate following CPR in elderly patients is lower than for younger people [28,29]. Although age factors were adjusted for in our logistic regression model, the results of this study may be problematic when applied to other countries with younger population compositions. However, our results will be useful for informing health policy makers in many developed countries with similar emergency systems and demographic profiles.

Third, we did not have data on the hospitals to which patients were transferred, meaning that the data did not reflect the quality of the hospital at which treatment was received. A recent study revealed that treatment at critical care medical centers was associated with better outcomes in cardio pulmonary arrest patients [30]. This may have also acted as a potential confounder.

Despite these limitations, our data provide a valuable investigation of almost all cases of OHCA subjects in Japan over a four-year period, constituting the largest-scale study of this issue to date.

## Conclusions

Our analysis of one of the largest samples of OHCA patients, including cases of cardiac and non-cardiac origin, revealed that shorter collapse-to-EMS CPR intervals were associated with better outcomes. Both one-month survival and neurologically favorable one-month survival curves against collapse-to-EMS CPR interval indicated that improving OHCA outcomes requires interventions to move the curve leftward (by shortening the response time) and upward (by improving the quality of CPR). Improving the emergency medical system, and the speed and quality of CPR in cases of OHCA are the key methods for improving the outcomes of OHCA.

## Key messages

● A nationwide HCA patient registry in Japan confirmed that shorter collapse-to-EMS CPR intervals were associated with better outcomes

● The logarithmic regression equation for the relationship with one-month survival was y = -0.059 ln(x) + 0.21, and that for the relationship with neurologically favorable one-month survival was y = -0.041 ln(x) + 0.13

● The logistic regression analysis after adjusting for potential confounders showed that longer collapse-to-EMS CPR intervals were associated with lower rates of one-month survival (OR 0.93, 95% CI: 0.93 to 0.93) and neurologically favorable one-month survival (OR 0.89, 95% CI 0.89 to 0.90)

● Improving the emergency medical system, and the speed and quality of CPR in cases of OHCA are key measures for improving the outcomes of OHCA

## Abbreviations

ACLS: advanced cardiac life support; AED: automated external defibrillator; BLS: basic life support; CI: confidence interval; CPC: cerebral performance category; CPR: cardiopulmonary resuscitation; ELSTs: emergency life-saving technicians; EMS: emergency medical service; OHCA: out-of-hospital cardiac arrest; ROSC: return of spontaneous circulation; SD: standard deviation; Vf: ventricular fibrillation; VT: entricular tachycardia.

## Competing interests

The authors declare that they have no competing interests.

## Authors' contributions

SK and TI jointly conceived and designed this study. TO conducted data cleaning. SK, TO, ST, MA, HY, HH, SM and TI jointly analyzed and interpreted the data. SK drafted the manuscript. All of the authors jointly reviewed and discussed the manuscript and revised it critically for important intellectual content and approved the draft for submission.
